# Engaging Community-Based Organizations to Address Barriers in Public Health Programs: Lessons Learned From COVID-19 Vaccine Acceptance Programs in Diverse Rural Communities

**DOI:** 10.1089/hs.2023.0017

**Published:** 2023-09-27

**Authors:** Dorothy Evans, Corina Norrbom, Spring Schmidt, Rachel Powell, Jacqueline McReynolds, Turquoise Sidibe

**Affiliations:** Dorothy Evans, MPH, MEd, is a Program Manager, Response Crisis, and Preparedness Unit, CDC Foundation, Atlanta, GA.; Rachel Powell, PhD, MPH, is Senior Program Manager, Response Crisis, and Preparedness Unit, CDC Foundation, Atlanta, GA.; Turquoise Sidibe, MPH, is Associate Vice President, Response Crisis, and Preparedness Unit, CDC Foundation, Atlanta, GA.; Corina Norrbom, MD, is an Assistant Professor, Medical College of Wisconsin-Central Wisconsin; a Health Policy Fellow, Wisconsin Institute for Public Policy and Service; and Project Director, Hmong and Hispanic Communication Network; all in Wausau, WI.; Spring Schmidt is Executive Director, Missouri Center for Public Health Excellence, and a PhD Candidate and Director, Office of Public Health Practice, St. Louis University College for Public Health and Social Justice, St. Louis, MO.; Jacqueline McReynolds, MHA, is Owner, Impact Advantage LLC, West Plains, MO.

**Keywords:** COVID-19, Rural health, Community-based organizations, Mistrust, Political polarization

## Abstract

Factors such as geography, community hesitancy, the political landscape, and legislative efforts to limit public health authority have contributed to a disproportionate number of COVID-19 infections and deaths in US rural communities. Community-based organizations are trusted entities that provide social and educational services in the communities where they live and have proven to be effective public health partners in response to the COVID-19 pandemic. Recognizing the unique challenges faced by rural communities, coupled with higher rates of vaccine hesitancy, the CDC Foundation awarded grants to 21 community-based organizations serving rural communities in 7 Midwest states to support the equitable uptake and distribution of COVID-19 vaccines. In this case study, 2 grantees, the Missouri Center for Public Health Excellence and the Hmong American Center, provide case studies that document their experiences, challenges, and strategies for overcoming barriers during the implementation of COVID-19 vaccine acceptance projects in diverse rural communities. These case studies provide key lessons learned that can be applied to future public health emergency and nonemergency responses to ensure that all members of communities are served well and protected.

## Introduction

Rural communities in the United States have experienced disproportionately high levels of COVID-19 infections and deaths. As of September 22, 2022, the cumulative rate of COVID-19 deaths was 396.7 per 100,000 population in rural (nonmetropolitan counties) counties and 290.71 per 100,000 population in metropolitan counties.^1^ Despite these high mortality rates, from December 14, 2020, to January 31, 2022, rural counties had lower first-dose vaccination coverage (58.5%) than urban counties (75.4%).^2^ Numerous reports, articles, and data have been published about the rural–urban divide in relation to COVID-19 vaccine acceptance and uptake. For example, a report from the US Centers for Disease Control and Prevention (CDC)^2^ and an article by Sehgal et al^3^ suggested that factors such as demographics, culture, and political affiliation impact an individual's attitudes toward vaccination. While many studies are doing the important work of collecting public health data and identifying potential sources for vaccine hesitancy, fewer studies have examined how collective behaviors might impact the public health response to mitigate the spread of COVID-19.

Throughout the COVID-19 pandemic response, the CDC Foundation—an independent nonprofit and the sole entity authorized by Congress to mobilize philanthropic and private-sector resources to support CDC's critical health protection work—has observed that community-based organizations (CBOs) are effective, trusted partners who serve in the communities where they operate. CBOs are public or private nonprofit organizations that include faith-based organizations, community coalitions, community development corporations, and nongovernmental social service agencies. Typically, CBOs are located in the communities they serve and led by people from the community. Strengthening the capacity of CBOs to engage in the COVID-19 response and promote vaccination helps authentic community voices to be heard and represented, which increases trust and helps ensure a more equitable, locally driven approach to ending the pandemic.

Since March 2021, the CDC Foundation's Response Crisis and Preparedness Unit has granted more than $12 million to 114 CBOs for COVID-19 vaccine acceptance projects in the most disproportionately impacted communities across 43 states. The projects engage with local partners and their communities to address vaccine-related concerns, develop innovative and culturally appropriate communications strategies, and promote timely vaccination. Project activities have included conducting educational campaigns (TV, radio, social media), hosting in-person and virtual community events, canvassing door-to-door, hosting health fairs and vaccination clinics, providing translation services, and staffing vaccine hotlines in multiple languages. In their narrative progress reports to the CDC Foundation, CBO grantees reported reaching more than 14.8 million individuals with COVID-19 safety and vaccine education messaging, administering 288,197 COVID-19 vaccinations within their communities, and partnering with 416 public health jurisdictions and 853 community organizations as of June 23, 2022.

Recognizing the unique healthcare and public health infrastructure challenges faced by rural communities, coupled with higher rates of vaccine hesitancy, the CDC Foundation—with support from private donors—awarded grants to CBOs serving rural communities in 7 midwestern states to support COVID-19 vaccine acceptance. The 21 CBO grantees that were selected served rural communities in Illinois, Indiana, Iowa, Michigan, Minnesota, Missouri, and Wisconsin. Grants were awarded in May 2021 and all grant activities ended by June 31, 2022.

Early in the implementation of the COVID-19 vaccine acceptance projects, many grantees highlighted in their internal reports the challenges and barriers related to individuals or institutions within the community. These included:

- **Lack of buy-in from elected officials and institutions and lack of engagement with potential partners.** “In many cases, [our organization] continues to encounter no response to our inquiries or have been greeted with potential partner hesitancy who share their reluctance to [be] perceived in their community as sponsoring a vaccine event.”
- **Disinterest or lack of capacity from health departments to support vaccination efforts and partnerships.** “Our challenge will always be our local health department because they don't want to field the calls of the community members asking questions. So, we established a Spanish vaccination/testing hotline for our county, and we are providing bilingual weekend vaccination events. We are collaborating with the state and recruiting our own bilingual staff to keep strain off the local health department.”
- **Pushback from the community.** “Individuals have received mixed messaging about COVID-19 and vaccinations and cling to their beliefs. These individuals do not respond well to having COVID-19 information pushed their way.”

These challenges, along with other issues impacting rural communities such as geographical isolation and distance or availability of health services, created a difficult environment for many of the CBO projects whose goals were to address the disproportionate impacts of the COVID-19 pandemic on rural communities. Two CBO grantees, the Missouri Center for Public Health Excellence (MOCPHE) and the Hmong American Center (HAC), experienced issues related to the rolling back of public health authorities' efforts, legal challenges, impacts of litigation during the public health emergency, political polarization, disinformation, and mistrust as impediments to effective risk communication and public health responses. In this case study, we present examples from these 2 grantee organizations that document their experiences, challenges, and strategies to overcoming barriers during the implementation of COVID-19 vaccine acceptance projects in rural communities.

## Case Studies

### Missouri Center for Public Health Excellence

MOCPHE is dedicated to improving public health services in Missouri. As a nonprofit membership organization, membership is open to the state's 114 local public health agencies (LPHAs) and public health system partners, such as universities and healthcare partners committed to creating the change necessary to build a stronger public health system in Missouri. LPHAs operate independently of each other and have unique relationships with state and federal public health agencies. They work directly with the Missouri Department of Health and Senior Services through contracts to deliver public health services to the communities they serve. For its COVID-19 vaccine acceptance project supported by the CDC Foundation, MOCPHE provided technical assistance to 12 LPHAs in rural communities in the forms of community outreach support, social media development, storytelling training, and health equity training. These technical assistance services were provided in response to an online survey that MOCPHE sent to participating LPHAs to identify their top needs related to the COVID-19 pandemic.

#### Legal Challenges Weaken Public Health Authority

LPHAs receiving support from MOCPHE expressed that a lack of trust—in CDC, the government in general, public health agencies, the COVID-19 vaccine, or the information being circulated—was the primary factor in vaccine hesitancy across all counties. A major barrier to addressing issues of vaccine hesitancy and mitigating the spread of COVID-19 in Missouri was the ruling by a Cole County Circuit judge on November 23, 2021, which determined that health orders issued by local health departments that were designed to stop the spread of COVID-19 violated the Missouri Constitution.^4^ Adding to the impact of the Cole County ruling was a communication from the Missouri Attorney General stating that “all mask mandates, quarantine orders, and other public health orders that are based on any of the invalidated regulations or issued outside the protection of the Missouri Administrative Procedure Act are null and void. You should stop enforcing and publicizing any such orders immediately.” The judge's decision was not appealed by the attorney general.

In the aftermath of the Cole County ruling, numerous LPHAs and local organizations halted elements of their response to the pandemic and other public health activities. Below are several specific examples:

- In November 2021, St. Clair County was investigating an outbreak of respiratory syncytial virus in a daycare facility. The investigation was halted after the Cole County ruling, despite the outbreak being unrelated to COVID-19.^5^
- Jefferson County halted issuing a quarantine order at a nursing home facility; 2 residents at that facility later died, possibly due to the lack of adherence to COVID-19 protocols.^5^
- McDonald County halted all COVID-19 orders, including isolation and quarantine requirements, and stated “we have no other option but to follow the orders of the Missouri Attorney General at this time.”^6^
- On December 13, 2021, in the absence of additional guidance from the state health department on the ruling, Bates County reported that the department would no longer conduct contact tracing or report metrics for COVID-19.^7^

The Cole County ruling also impacted school districts across the state; the Attorney General's office sent cease and desist letters to numerous school districts warning that they did not have the authority to issue “mask mandates, quarantine orders, or other public health orders” and “failure to follow the court's judgment may result in enforcement action against you to remove orders the court has determined are unconstitutional and illegal.”^8^

In the aftermath of the ruling, some local legislative bodies voted on whether to keep their mask mandates; Jackson County rescinded theirs, as it would set them apart from other, neighboring counties who were no longer requiring masks.^9^ St. Louis County also rescinded their mask mandate after the St. Louis County Council failed to secure majority support for the mandate.

Larger jurisdictions with available financial, political, and legal resources interpreted these legal challenges in their own way and acted according to their own sense of comfort and security in either following or fighting through these issues. However, those resources are not equitably distributed. Most of the LPHAs in Missouri are independent government agencies who exist outside of their own county/jurisdictional governments. They do not always have the same levels of access to other municipal resources, including legal counsel, fiscal services, or other structural support mechanisms. Given that Missouri also consistently trails last in per capita public health spending across the nation, that could mean that smaller, rural departments may lack the necessary resources to address these legal threats effectively.

In addition to the disruption and uncertainties created by the complicated legal environment, competing narratives in the public discourse framing public health created confusion, miscommunication, and mistrust among large segments of the population. Differing priorities and interpretations of core public health messaging have exacerbated issues that are common pressure points related to state versus local rule, urban versus rural differences, or stratified opinions based on socioeconomic status, political affiliation, or racial/ethnic identity. Given some of the identified issues, there may also be financial, legal, and political barriers to creating effective communication campaigns in rural environments, particularly when the prevailing state narrative from public officials opposes a wide array of potential public health interventions. LPHAs in rural communities are often strong community partners with deep ties directly to residents. However, the lack of resources combined with counternarratives that have full media attention, wide routes of dissemination in communities, and that are targeted at local public health officials can create system breakdowns where public health services suffer.

#### Successful Provision of COVID-19 Information

**Figure 1.**
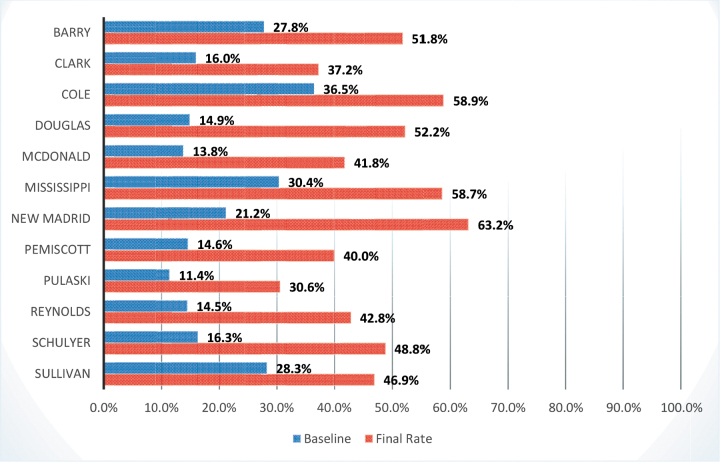
Increases in COVID-19 vaccination rates between June 25, 2021, and March 7, 2022, in each of the 12 participating Missouri counties. Data from the Missouri Center for Public Health Excellence Program Evaluation Data.^10^

The overall goal of MOCPHE's project was to increase COVID-19 vaccination rates in each of the 12 participating counties by 25% from baseline, from June 25, 2021, to March 7, 2022 (Figure 1). Directly attributing an increase in vaccination rates to the work of this project would be difficult. However, it is evident from participant feedback surveys completed by LPHA staff that technical assistance provided by MOCPHE was beneficial. For example, the development of a community outreach campaign called “7 in 7”—based on the CDC guidance that people need to hear consistent information from 7 different sources before making behavior change—led to increases in consistent and credible COVID-19 messaging outside of the political barriers people were facing. In this campaign, participating LPHAs were encouraged to ask 7 community partners to share credible COVID-19 materials and messaging for 7 weeks. LPHAs engaged organizations such as the Chamber of Commerce, banks, academic institutions, the Office of Economic Security, community center, electric company, and other community partners. By partnering with trusted community institutions, LPHAs successfully provided COVID-19 and vaccine safety information during a time when their authority to carry out public health services were being challenged in the state. This partnership model could be applied to other public health emergencies, and local agencies could benefit by receiving additional resources and support as a result of their partnership with a community organization.

#### Lessons Learned and Opportunities to Improve

Lessons learned from implementing a COVID-19 vaccine acceptance project with 12 Missouri LPHAs and opportunities to improve future public health emergency preparedness and responses include the following:

- All LPHAs should have access to legal representation that is specific to their department. Many LPHAs do not have access to a lawyer for research, advice, or representation on how changes in court rulings or legislation affects them. Many of the actions of a department are grounded in statute, rules, or regulations, but only consistent legal support can provide the expertise necessary to understand the impact on local operations when there are challenges or changes to their understanding or interpretation of the legal underpinnings of their actions.
- Public health authority and the underlying basis for it should be required educational content for all levels of local and state government. Many of the challenges to authority related to COVID-19 orders had a much wider effect than intended on the conduct of other public health activities. A “whole-of-government” approach to some public health actions is worth exploring in some areas where there is insufficient support for the individual LPHA.
- Public health associations can play a powerful role in navigating statewide political issues and leveraging the voices of multiple departments and community leaders to generate the necessary education and impact for change, especially at the legislative level. Additional resources that supported the ability of LPHAs to participate in effective associations can be a helpful strategy in building system-level solutions that affect more than 1 jurisdiction.
- LPHAs need significant support for communication infrastructure, personnel, and access to expertise to deliver high-quality messaging that is tailored to their jurisdictions. Many local agencies have strong community partnerships and will use effective communication products and techniques if they can be made available. MOCPHE recommends establishing an infrastructure that creates shared communication resources accessible to multiple rural health departments to manage misinformation and rebuild public health infrastructure. The combination of strong community relationships and high-quality communication materials can have a direct impact on health outcomes.

### Hmong American Center

Immigrant communities are particularly vulnerable to the negative health, educational, and economic impacts of COVID-19. Challenges include language differences, cultural and literacy barriers, insufficient awareness of available services and resources, historical trauma and mistrust, lack of protection in workplaces, differences in health beliefs, immigration issues, and discrimination. Anchor institutions and local governments in central Wisconsin were not prepared or equipped to reach immigrant communities to prevent and mitigate the spread of COVID-19.

Central Wisconsin is largely rural, and the US census has documented major demographic shifts over the past 40 years. Marathon County was 99.8% White in 1975, but there are now more than 12,000 Southeast Asian residents in Marathon and adjacent counties.^11^ As of October 2018, the City of Wausau (the county seat of Marathon) had the highest per capita Hmong population in Wisconsin (approximately 12%)^12^ and remains among the top 10 US metropolitan areas with the highest Hmong population.^13^ In the same 8-county area there is also a growing Hispanic population, particularly in agricultural areas, with nearly 14,000 Hispanic people according to 2020 census data, and substantial numbers of undocumented individuals.^14^ Some 70% of elementary school children in 1 rural school district are Hispanic.

#### Hmong and Hispanic Communication Network Partnerships

In response to existing communication barriers with Hmong and Hispanic communities in central Wisconsin, the Wisconsin Institute for Public Policy and Service (WIPPS) convened community partners in March 2020 to envision and develop what became known as the Hmong and Hispanic Communication Network (H2N). A unit of the University of Wisconsin System based in Central Wisconsin, WIPPS developed relationships and a reputation for bringing people and organizations together from diverse perspectives for civil discourse and to achieve common goals. The network included Hmong and Hispanic community leaders, a medical school, health systems, resource agencies, and county public health departments. WIPPS was able to raise money through a variety of small grants and became the backbone organization for the project. A major partner was the HAC, central Wisconsin's only Hmong CBO. With capacity-building assistance from WIPPS, the HAC became the fiscal agent and hub for H2N in spring 2021 and the recipient of a CDC Foundation COVID-19 vaccine acceptance grant.

H2N focused on strengthening communication channels and facilitating regular information exchange between public health, health systems, and resource agencies and people in Hmong and Hispanic communities. The model consistently incorporated the insights of Hmong and Hispanic community members and regular collaboration with community organizations. H2N envisioned bidirectional communication not only for the pandemic, but as part of a longer-term solution to address health equity. Community health workers (CHWs) were identified as a solution that could help build trust, although CHWs did not exist in a formal way in Central Wisconsin before the pandemic.

H2N Hmong and Hispanic community coordinators hired CHWs who participated in COVID-19 prevention and mitigation trainings, including modeling prevention strategies such as wearing masks, physical distancing, hand washing/sanitizing, and eventually vaccinations, which prepared them for the CDC Foundation vaccine acceptance grant work.

One-on-one conversations with trusted partners were critical for health education and outreach. CHWs conversed with their extended families and social circles. They talked with people at food distribution events, farms, churches, festivals, community centers, ethnic grocery stores, and food processing plants. CHWs met with small town community leaders and farm and business owners to create opportunities to meet the people they were trying to reach. H2N CHWs had more than 8,000 conversations in their communities between May 2020 and spring 2022. Additionally, CHWs recorded public service announcements, held live programs on Hmong and Hispanic radio, and developed messaging for YouTube^15^ and other social media platforms in Spanish and Hmong to address myths and answer common questions.^16^ To foster relationships and gain trust, CHWs had to be sensitive to immediate community needs and connect people with resources such as rental assistance, food, health insurance and healthcare access, legal advice, and educational opportunities.

#### Barriers Contributing to Vaccine Inequities

Early 2021 saw significant COVID-19 vaccination inequities that impacted Hispanic and Hmong communities. One barrier leading to inequity was that many Hmong and Hispanic people were not connected with a health system. In addition, registration for COVID-19 vaccination was generally done online (with websites written only in English), which was problematic given language and literacy barriers and often unreliable or limited internet access. Misinformation circulated, and there was a lack of official information broadcast on Hmong and Hispanic media outlets. The public charge rule and other immigration fears kept people away from institutions they did not trust. Challenging work schedules, transportation deficits in rural areas, and the effects of immigration status on one's ability to apply for a driver's license were further barriers.

Given these barriers, most people accessing limited COVID-19 vaccines were non-Hispanic White. H2N and the HAC were persistent in advocating for designated vaccination slots for Hmong elders and Hispanic people with chronic diseases or other risk factors for severe COVID-19. Health departments were not familiar with—nor did they have the capacity to address—barriers impeding vaccine-eligible Hmong and Hispanic people from accessing vaccines. After consistent advocacy, 25 vaccination slots were allotted specifically to Hmong elders if the HAC recruited people, scheduled the vaccination, and organized transportation to the health department. Unfortunately, slots were not designated for people from the Hispanic community.

Health departments declined H2N requests to partner on community-based pop-up clinics, citing logistical complications and insufficient staff. Health departments relied on people coming to them and on the established health systems to distribute the majority of vaccinations. When a task force was set up by the health department to develop a strategy for vaccinating Black, Indigenous, and communities of color, the H2N project director pointed out that not a single Hmong or Hispanic person was at the table. It also became apparent that health systems focused on serving their paying patient population, with 1 system explicitly saying they were vaccinating their own patients first.

#### Health Promotion Efforts to Improve Vaccine Acceptance

Initially, CHWs assisted vaccine-eligible people with finding and registering for vaccinations. When vaccination eligibility opened, H2N began working with partners to organize pop-up clinics in the community in safe, convenient locations at times amenable to varying work schedules. CHWs recruited and welcomed participants at these clinics, answered questions, and reassured people in their preferred language and in a culturally sensitive way. The H2N team eventually broadened the focus of its vaccination clinics to be community and family events—fun mixed with health promotion. Additionally, COVID-19 test giveaways and demonstrations, education from health navigators, and blood pressure checks were offered. Between December 2020 and June 2022, H2N held 50 pop-up vaccination clinics at farms, community centers, schools, churches, and festivals, administering 1,253 COVID-19 and more than 500 influenza vaccinations to underserved communities. H2N's COVID-19 interventions remain deeply intertwined with related activities to advance health equity.

#### Lessons Learned and Opportunities for Improvement

Lessons learned from implementing a COVID-19 vaccine acceptance project among diverse communities in rural Wisconsin and opportunities to improve future public health emergency preparedness and responses include the following:

- **Improving transparency, establishing relationships, and maintaining trust** are important during an evolving health emergency. CHWs are the eyes, ears, and voices for their communities. CHWs based in a CBO were able to be more nimble to adapt and innovate. Although there are effective models for CHWs based in health departments or health systems, being employed by a CBO seemed to empower not only the CHWs, but also their communities.
- **Paying CHWs fair wages for their work** was a way to push back on the assumption by certain people in power that people of color should be willing to represent their communities and provide services for free. Providing CHWs with continuing educational and networking opportunities through grantee meetings, conference presentations, local advisory boards, and other capacity-building efforts contribute to developing leaders. Several CHWs went on to get full-time jobs with the University of Wisconsin and the Wisconsin Department of Health Services where they can advocate for advancing health equity at upstream and policy levels.
- **Increasing the number of diverse community voices at decisionmaking tables** in appointed and elected, public and private boards, and other positions of power. H2N repeatedly had to bring disparities to light to ensure that Hispanic and Hmong community coordinators were included in conversations. Institutions should proactively engage with and recruit individuals representing diverse communities to participate on local boards or other decisionmaking bodies.
- **Partnering anchor institutions with local organizations**, such as CBOs, to support culturally accepted public health responses. Anchor institutions, including health departments and health systems, were ill-prepared to communicate with people who did not speak English, read, listen to local TV stations, or visit public health or health system websites. They were not prepared to handle the historical trauma of refugees or religious and cultural disease constructs that do not include viruses.

## CBO Participation Is Crucial

To complement the governmental public health response and to ensure the equitable distribution and uptake of COVID-19 vaccines, especially in communities that are under resourced, the CDC Foundation partnered with CBOs serving rural communities to provide education and access to vaccines. Overall, CBOs are a protective factor for communities—they have their finger on the pulse of the community's needs, concerns, and assets. CBO staff are usually representatives of the communities they serve and are likely to have an established deep trust and connection to the community. As an extension of the public health system during the COVID-19 pandemic, CBOs faced many of the same issues related to pushback and hesitancy as governmental health departments. The challenges faced by MOCPHE and H2N highlight many of the complex political and social factors that undermine the delivery of public health services and threaten the health of rural communities as well as the existing power dynamics between organizations. Public health preparedness plans can be strengthened by ensuring that CBOs' perspectives are included, especially in communities where disparities are present. The inclusion of CBOs is critical for addressing current issues and planning for the next emergency response.

## Conclusion

During the COVID-19 pandemic, the political landscape, pushback from community, and legislative efforts to erode public health authority also impacted CBOs, especially those serving under resourced rural communities. Distrust of institutions rapidly grew and undermined the work of informing and protecting families and communities during a pandemic. If governmental public health agencies and nonprofits, such as CBOs, continue to face these challenges to providing the most basic health and safety services, disparities in the distribution of resources and negative health outcomes for individuals living in rural communities will likely grow. Lessons learned include involving diverse community representatives from the beginning in shaping the plans of local public health agencies and relying on CBOs as trusted resources to fill in gaps when state or local policies are limiting and/or erasing the opportunity for public health agencies to serve the public. These key lessons should be applied to both emergency and nonemergency public health work to ensure that all members of communities are served and protected.
